# Continuous culture of *Escherichia coli*, under selective pressure by a novel antimicrobial complex, does not result in development of resistance

**DOI:** 10.1038/s41598-019-38925-9

**Published:** 2019-02-20

**Authors:** Lilit Tonoyan, Gerard T. A. Fleming, Ruairi Friel, Vincent O’Flaherty

**Affiliations:** 1 Microbiology, School of Natural Sciences and Ryan Institute, National University of Ireland Galway, Galway, Ireland; 2 Westway Health, Unit 120, Business Innovation Centre, National University of Ireland Galway, Galway, Ireland

## Abstract

We attempted to generate *de novo* resistance to a newly described biocidal complex, ITC (iodo-thiocyanate complex), and to levofloxacin (LVX) in *Escherichia coli* ATCC 25922, by means of selective chemostat culture. We measured resistance by determining the minimum inhibitory concentrations (MICs) for these agents. *E. coli* underwent 20-day parallel adaptive evolution routes under no antimicrobial selection, and gradually increasing ITC and LVX selection pressure. Long-term exposure of *E. coli* to ITC did not induce resistance to ITC, or cross-resistance to LVX. No distinct mutational pattern was evidenced from whole-genome sequence (WGS)-based comparisons of ITC-challenged and unchallenged bacterial populations. Moreover, the exposed *E. coli* population could not survive a 2 × MIC challenge of ITC. By contrast, resistance to LVX was rapidly induced (on day 1 the MIC had increased 16-fold), selected for (by day 14 the MIC had increased 64-fold) and enriched with a highly characteristic genome mutational pattern. WGS of this evolving population revealed that the majority of mutations appeared in the genes of LVX target proteins (GyrA, ParC, ParE) and drug influx (OmpF). This study suggests that the usage of ITC may not trigger the emergence of facile resistance or cross-resistance, in contrast to common antibiotics.

## Introduction

Antibiotic resistance is a major global health problem. Preventing the emergence of resistance requires a thorough understanding of the underlying causes. Mechanisms of bacterial resistance to antibiotics, or other antimicrobial agents, have been well documented. Some bacteria possess intrinsic resistance towards antibiotics as natural structural and functional properties, while many others acquire resistance, mediated by several biochemical mechanisms (e.g. to stop its entrance, to pump it out, to break it down and to inactivate its target), by gene sharing between bacteria, or *de novo* as a result of specific gene mutations (spontaneous or induced by an agent)^1^. Though development of antibiotic resistance is a natural phenomenon and inevitable, the emergence of widespread, multi-agent resistance in many bacteria, including important pathogens, has been accelerated by the use, misuse and abuse of antibiotics and other antimicrobial agents, including triclosan, triclocarbon and others^2^. Preservation of antibiotics, underpinned by the availability of novel antimicrobial biocides that avoid the emergence of antibiotic cross-resistance, is thus an important societal goal.

We recently described a new biocidal complex, which we named the iodo-thiocyanate complex (ITC), and reported on its antibacterial properties^3^. In parallel, we assessed the *in vitro* cytotoxic, hemolytic and genotoxic potential of ITC by comparison with common antiseptics, which suggested the feasibility for use of ITC as a biocide/antiseptic^4^. Generally, a biocide is described as a chemical agent, usually broad-spectrum, that inactivates microorganisms^5^. The term biocide is mainly defined by its usage^6^ and includes disinfectants, antiseptics and preservatives^7,8^. Biocides generally have multiple targets in the bacterial cell and the cell death is caused by combined detrimental effects^9^. Moreover, the target sites can differ according to the biocide concentration applied and interactions resulting with cell death are not always clearly defined^10^. Our study showed that ITC is an oxidative mixture containing reactive oxygen (ROS) and iodine species that possess biocidal properties^3^. These reactive species appeared to interact with bacterial DNA and ribosomes, however, like other biocidal agents, the exact cause of cell death induced by ITC was difficult to pinpoint. Taking into account that such reactive species provide direct and indirect mechanisms for mutagenesis^11^, the potential for the development of bacterial resistance toward ITC, and other antimicrobials, is of concern in the context of its potential application. Indeed, there is increasing concern that extensive biocide usage can be responsible for the selection and maintenance of antibiotic-resistant bacteria in various environments^12^. Accumulated experimental evidence suggests that bacteria that have become less sensitive to a biocide (because of exposure to the biocide) also show decreased susceptibilities to antibiotics. Household pine oil cleaner (POC) resistant mutants of *Staphylococcus aureus* isolated on media containing POC also demonstrated reduced susceptibility to the cell wall-active antibiotics vancomycin and oxacillin^13^. The sequential growth of *Salmonella typhimurium* in gradually increasing concentrations of triclosan in the growth medium resulted in a population that was highly resistant (2000-fold) to triclosan and 8-fold resistant to ampicillin^14^. Population of *Pseudomonas aeruginosa* subjected to increasing levels of benzalkonium chloride (BKC) selection pressure in long-term continuous culture carried a mutant variant with >12-fold decreased sensitivity to BKC and 256-fold increased resistance to fluoroquinolone antibiotic ciprofloxacin^15^.

In recent years, substantial research efforts have been directed towards understanding bacterial adaptation and resistance to different types of biocides^16^. As the modes of action of biocides are generally difficult to distinguish, the detailed mechanisms underpinning acquired bacterial resistance to biocides also remain uncertain^12^. From the genetic point of view, biocide resistance can arise *via* mutation or by the acquisition of genetic elements bearing resistance genes. From the mechanistic point of view, because of the multiplicity of target sites, resistance to biocides is less common acquired *via* target inactivation mechanisms. More commonly, biocide resistance can be developed due to the inactivation of the biocide and the reduction of its intracellular concentration to a level that is not harmful to the bacterial cell, that is to say, reduced influx, or enhanced efflux of a biocide^8,17,18^. Considering the common shared mechanisms for resistance towards antibiotics and biocides, there is a likelihood of cross-resistance between biocides and antibiotics.

Since resistance is a response adaptation of bacteria to strong natural selection imposed by the application of antibiotics and biocides, understanding the adaptive processes in pathogen populations and, in particular, characterizing the genetic changes that turn drug-sensitive bacteria into resistant variants, is essential to address the problem of resistance. Laboratory evolution experiments have been successfully used to study pathogen adaptation to antibiotics during the evolutionary process, in real time and under precisely controlled conditions^19^. Chemostat-based continuous flow systems are one of the generally applied experimental evolution techniques^20^. In a chemostat vessel, the bacterial population can be maintained in exponential growth phase continuously by the regular inflow of fresh media and simultaneous outflow of waste culture^21^. Bacterial populations in these continuous cultures become highly heterogeneous in remarkably short time periods existing as a temporal, spatial, physiological and genetic heterogeneous complexity^22^. Although mutations in chemostat populations will occur naturally in the presence, or absence, of a mutagenic agent, their evolution may be “directed” by the addition of a selector (such as an antimicrobial drug) and the population may be enriched with a desired phenotype (such as antimicrobial-resistant)^23^. In such settings, the evolving pathogen population may be continuously challenged by a temporal gradient of drug concentrations to promote the sequential emergence and fixation of multiple resistance mutations leading to increasingly higher resistance levels^24,25^. Chemostat continuous culture systems may thus simulate bacterial evolution in the host environment, in response to an antimicrobial treatment. As would be expected at an infection site, the pathogens in continuous cultures are grown at high densities, contain physiologically refractory planktonic (persisters) and biofilm subpopulations and antimicrobials are supplied in a continuous manner^26^.

Microbial experimental evolution studies have been revolutionized by recent advances in nucleic acid sequencing technologies. It is now technically and financially feasible to associate the phenotypic response to the underlying genetic changes during an experimental evolution study. Combining experimental evolution with whole-genome sequencing of evolved populations may uncover the genetic basis of laboratory adaptation to antimicrobials and the emergence of antimicrobial resistance^27^. This approach may reveal how many mutations underlie antimicrobial resistance, how are they distributed across the genome (coding versus noncoding, synonymous versus nonsynonymous mutations) and through time (mutations rise and fix at early or later stages of adaptation) and which target genes contribute to resistance^28^.

In this study, we used enrichment chemostats in attempts to generate *de novo* resistance to the ITC biocide and to assess its relationship to cross-resistance to a bactericidal antibiotic in a model organism. *E. coli* ATCC 25922 was used as the model organism, as it is well-characterized at a molecular and physiological level and a complete annotated genome sequence is available. Levofloxacin was selected as the antibiotic of choice as increased resistance to LVX may be acquired in *E. coli* under chemostat settings^23^ and the mechanisms of resistance toward LVX are well documented. Starting with an isogenic drug-sensitive *E. coli* ATCC 25922, we exposed the evolving population to increasing levels of ITC or LVX. To prevent culture washout, the concentrations of the antimicrobials were increased in a stepwise manner, with increases applied when the density of the culture had almost completely returned to the pre-antimicrobial treatment level^15^. We monitored the development of ITC and LVX-resistant phenotypes by changes in minimum inhibitory concentration profiles. In parallel, we followed ITC and antibiotic resistance emergence in unexposed (control) populations. Next, we performed WGS analysis of planktonic polymorphic populations sampled over time and compared starter, intermediate and final populations to reveal possible mutational determinants of ITC and LVX resistance.

## Results

### Growth and phenotypic resistance profiles of chemostat-derived cultures

The *E. coli* ATCC 25922 was gradually adapted to increasing concentrations of ITC and LVX over 20-days of selective chemostat culture, with the aim of enriching the population with antimicrobial-resistant bacteria. The emergence of drug resistance in these exposed cultures was then compared with unexposed culture.

**Figure 1:**
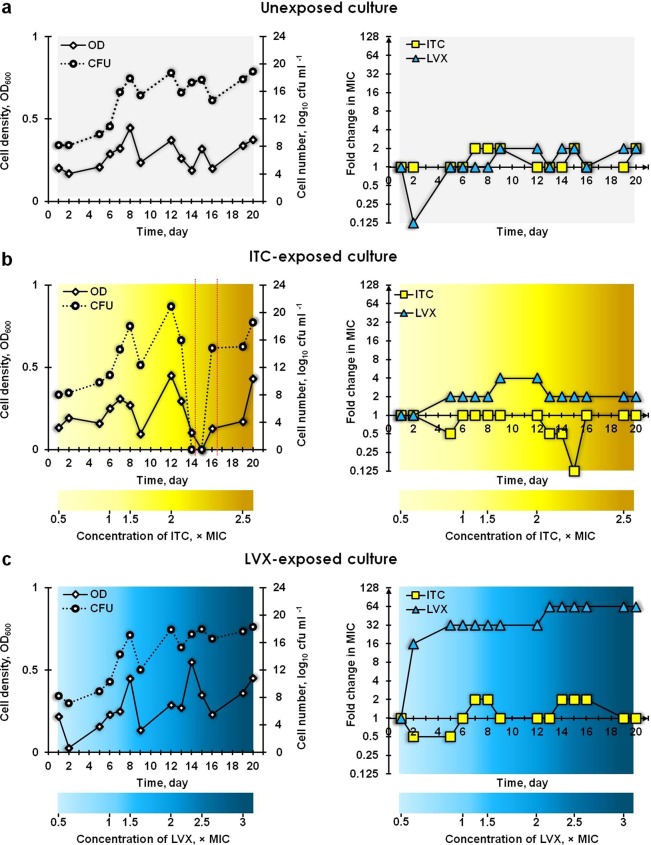
Growth and MIC profiles of continuous cultures of *E. coli* ATCC 25922: (**a**) control unexposed, exposed to (**b**) ITC and exposed to (**c**) LVX. The optical density (OD; 600 nm), viable cell counts (colony forming units; cfu ml^−1^) and minimum inhibitory concentrations (MICs; µg ml^−1^) of ITC and LVX were measured daily for parallel populations evolving under no antimicrobial selection, selection with ITC and LVX. On the last day, the concentrations of ITC and LVX added to the medium reservoirs corresponded to 2.5× and 3× their original MICs, respectively. The fold change in MIC was the ratio of the MIC obtained on the particular sampling day to the MIC recorded on day 1. The area delineated with red dotted lines represent the batch state of ITC-exposed culture.

The growth and MIC profiles of three cultures are summarized in Fig. 1. The cell density and the number of cells fluctuated in the chemostat without added selection pressure (Fig. 1a). Such oscillations can be due to stochastic experimental measures or can be attributed to population-wide die-offs and recoveries^29^. The MIC values of ITC and LVX towards this unexposed continuous culture did not alter substantially between the first and last day (day 20) of cultivation (Fig. 1a; Supplementary Table S1). This indicated that the culture, which was not exposed to any antimicrobial agent, did not develop resistance toward the ITC and LVX during the 20 days of evolution.

The cell density and cell count measurements of the ITC-exposed culture (Fig. 1b) indicated that, despite the administration of a media containing 0.5 and 1 × MIC of ITC into the chemostat vessel, the *E. coli* ATCC 25922 grew exponentially up to day 8. The *E. coli* population declined in response to a 1.5 × MIC supplementation of ITC on day 8, but resumed growth during the following days. Subsequently, a 2 × MIC of ITC was applied into the vessel on day 12, which caused growth to abruptly cease – the culture density decreased progressively reaching 0 on day 15, while no viable cells were detectable on either day 14 or 15. On day 15, the feeding of the ITC-exposed culture was paused overnight. Following this, the culture resumed growth, possibly as a result of i) physiologically antimicrobial-refractory wall-adhering survivors reseeding the planktonic population and re-establishing a population which is as sensitive to antimicrobials as a naïve planktonic population^26^; and/or ii) physiologically antimicrobial-refractory non-dividing or slowly-dividing persisters re-growing a new population as sensitive to antimicrobials as naïve bacteria^30^. During the batch state (the sector delineated with red dotted lines; Fig. 1b), the ITC-exposed culture further revived to the density of the antimicrobial-free culture due to the cell-associated decline in the effective concentration of ITC in the culture^26^. As the 2 × MIC concentration of ITC waned, the bacterial population recovered after the preceding clearance. However, the recovered population was not as sensitive towards replenished 2 × MIC dose of ITC compared to the population preceding the clearance, as it did not encounter gradually increasing ITC stress over 13 days. In consequence, the culture outlived the 2 × MIC of ITC. Regarding the MIC profile, there was no increase in the ITC MIC in samples tested throughout the culture experiment, despite prolonged exposure to ITC (Fig. 1b; Supplementary Table S1). However, a decrease in MIC was noted on days 13, 14 and 15 compared to the samples tested on days 9 and 12, which may have been an artefact created as a result of culture decline and low starting inoculum density for MIC determinations. No increase was recorded either in the MIC of LVX in the ITC-exposed culture, although 4-fold fluctuations were noted on days 9 and 12. These observations indicated that exposure to ITC did not induce resistance to it, nor did it induce cross-resistance to LVX. In addition, ITC at a concentration of 2 × MIC was able to eliminate the planktonic bacterial population despite 13 days of prior exposure to ITC (days 14, 15; Fig. 1b).

On day 1, feeding the *E. coli* ATCC 25922 culture with medium containing 0.5 × MIC of LVX, inhibited the growth of the culture and had also triggered a 16-fold increase in LVX MIC by day 2 (Fig. 1c; Supplementary Table S1), indicating a rapid adaptation of the culture to LVX. Subsequently, the culture continued to grow exponentially and the MIC against LVX increased further (32-fold that of the inoculum by day 5), indicating that the culture was being enriched with more resistant variants. The subsequently applied stepwise challenge with LVX caused the culture density and cell number to fluctuate in a saw-tooth pattern – the introduction of a 2 × MIC LVX selected for higher resistance, leading to a 64-fold increase in LVX MIC by day 13 of culture. The MIC of ITC towards the LVX-exposed culture did not alter substantially during the experiment (Fig. 1c; Supplementary Table S1). These results indicated that a stepwise challenge of an *E. coli* ATCC 25922 culture, with increasing concentrations of LVX, rapidly selected for LVX-resistant phenotypes, failed to eliminate the planktonic population and did not induce cross-resistance against ITC.

### Genotypic profile of chemostat-derived cultures

We performed whole-genome sequencing on samples of the planktonic polymorphic populations isolated over the course of the chemostat selection experiments and determined the allelic frequencies of all mutations that reached at least 5% frequencies within an individual population. We sequenced, using the Illumina platform, the genomes of mixed populations from unexposed chemostat culture from day 1 (start), day 14 (intermediate) and day 20 (end), ITC-exposed culture and LVX-exposed culture on days 14 and 20, and then screened for mutations using the BRESEQ pipeline. Our principal focus was on data obtained from thawed cultures (denoted TC), but we also sequenced revived cultures (denoted RC) for quality assurance. Mutations identified as being shared among thawed and revived cultures are available as Venn diagrams in Supplementary Fig. S1.

**Figure 2:**
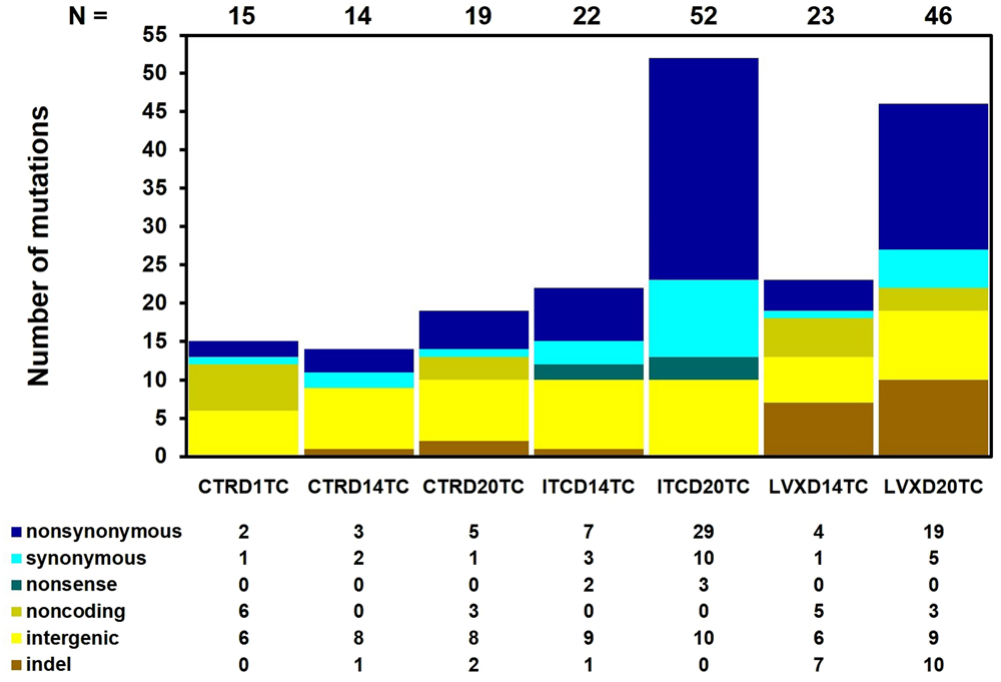
Mutational spectrum of chemostat-derived *E. coli* ATCC 25922 polymorphic population samples. “Metagenomes” of selection-free (CTR day 1, 14, 20), ITC-exposed (day 14 and 20) and LVX-exposed (day 14 and 20) thawed cultures were sequenced using the Illumina platform and polymorphisms were identified by BRESEQ. The total number of mutations (N) in each culture is shown above each column. The types of mutations present in each culture are presented below the columns. Base substitutions are presented as nonsynonymous (a point mutation altering the amino acid sequence of a protein), synonymous (a point mutation not altering the amino acid), nonsense (a point mutation resulting in a premature termination codon), noncoding (a mutation in noncoding RNA genes) and intergenic mutations; indels are deletions and insertions of ≤20 nt size.

**Figure 3:**
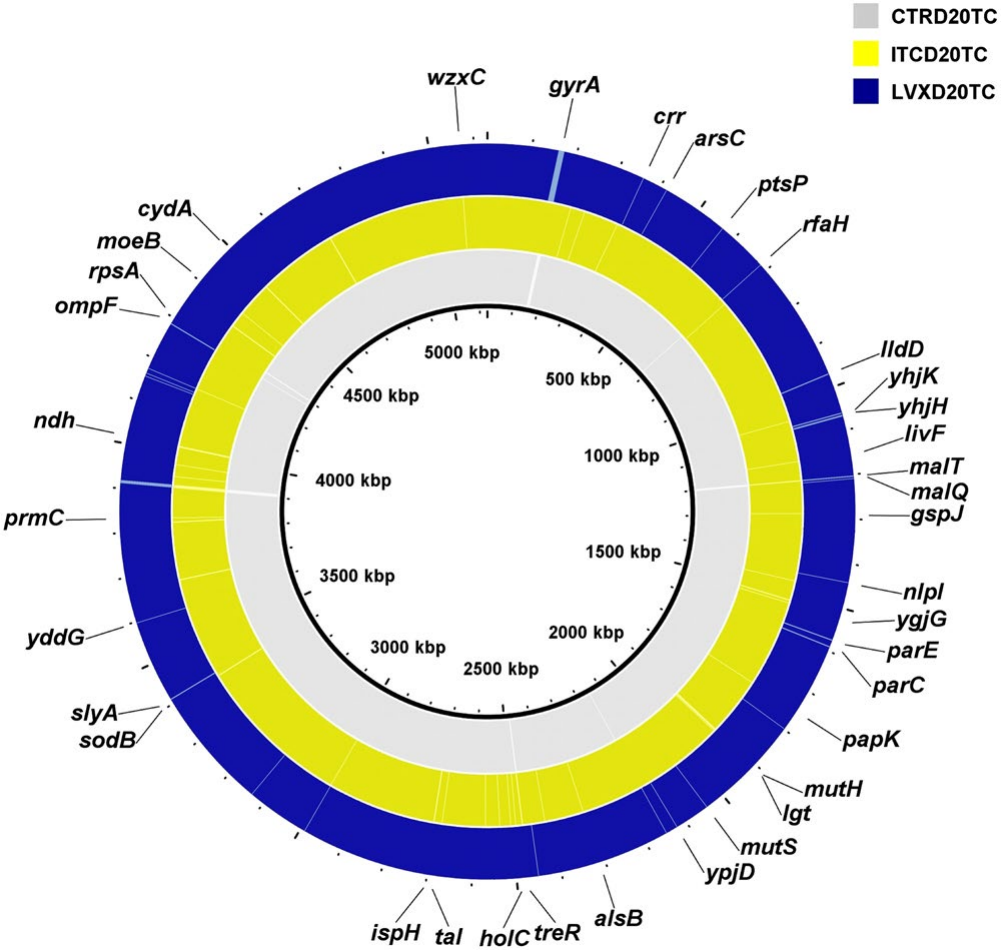
Mutations identified in chemostat-derived *E. coli* ATCC 25922 polymorphic populations. Genome visualization and comparison of final day unexposed, ITC-exposed and LVX-exposed populations to *E. coli* reference genome (CP009072) was performed using BRIG. From inner to outer rings: reference genome, unexposed thawed culture at day 20 (CTRD20TC), ITC-exposed thawed culture at day 20 (ITCD20TC) and LVX-exposed thawed culture at day 20 (LVXD20TC). The gaps shown indicate where the sequence data from chemostat populations differ from the reference sequence.

The total number and the type of genetic alterations identified in the chemostat-derived thawed cultures are shown in Fig. 2, mutations identified in thawed cultures at final day of evolution is presented in Fig. 3, while the detailed information on predicted mutations is provided in Supplementary Table S2. BRESEQ detected several large insertions and amplifications in the samples, but as all occurred in repeat regions, they were not considered in this analysis to avoid any concern about inaccurate short-read alignment. The number of total mutations in all treatment groups increased over time, reaching the highest totals in the LVX and ITC-exposed day 20 populations (Fig. 2). The counts of nonsynonymous substitutions also increased over time in all the test populations. This is important in that it shows the accumulation of random mutations with chemostat experiment.

The inoculum population, which was started from a single isogenic colony and was grown in a batch mode for 24 h (CTRD1TC) already bore a collection of mutations by comparison with the reference *E. coli* ATCC 25922 isolate (Fig. 2; Supplementary Table S2). The majority of these mutations occurred in noncoding (6/14), or intergenic regions (6/14). Most interestingly, two nonsynonymous single nucleotide polymorphisms (SNPs) were also observed. Notably, the CTRD1TC population was already polymorphic containing subpopulations with a common LVX conferring mutation at position 87 for the GyrA subunit of DNA gyrase. The same codon in *gyrA* was a subject to two mutations: D87G at 64.3% and D87H at 31.8% frequencies (Supplementary Table S2). These two mutations were also present in the unexposed population on days 14 and 20, however, their frequencies fluctuate, and we cannot predict whether the mutations would have ultimately been lost or retained. Similarly, the population revived from the unexposed day 1 culture (CTRD1RC) contained subpopulations bearing repeated *gyrA* D87G and D87H mutations at relatively similar frequencies (Supplementary Table S2). In any case, according to the MIC profile, the unexposed polymorphic population did not display or develop resistance towards LVX throughout the culture history (Fig. 1a; Supplementary Table S1). In addition, these two nonsynonymous *gyrA* D87 mutations were not present in the ITC and LVX-exposed populations on days 14 and 20. The unexposed culture on days 14 and 20 (CTRD14TC and CTRD20TC) had a slightly different set of mutations by comparison with day 1. Of interest, perhaps, are the nonsynonymous A221P substitution in *malT* gene (MalT, the transcriptional activator of the maltose regulon; Fig. 3; Supplementary Table S2), 6 nt deletion in *treR* coding (99–104/948 nt) region (TreR, the repressor of operons involved in trehalose transport and degradation under osmotic stress; Fig. 3; Supplementary Table S2) and an A → G substitution in the intergenic region between the dicarboxylate symporter family protein/inner membrane protein Alx (Supplementary Table S2). These changes were present only in the CTRD14 and CTRD20 samples (in both thawed and revived cultures), at >15% frequencies.

Although the ITC-exposed culture did not show decreased susceptibility towards ITC or LVX, we sought genomic alterations that could potentially contribute to the development of ITC resistance, or antibiotic cross-resistance. We examined the mutation list (Supplementary Table S2) of ITC-exposed populations to identify genetic determinants, which may represent possible mutational targets in this evolved population. In most cases, however, we do not know if a particular mutation inactivates, activates, or otherwise acts on the activity of the gene product. The *E. coli* ATCC 25922 population, which was under selection pressure through exposure to ITC, accumulated a higher number of nonsynonymous and synonymous SNPs over time compared to the other cultures (Fig. 2). The frequencies at which these mutations occur in the ITC-exposed populations are, however, generally low (Supplementary Table S2). Distinctively, the ITC-exposed populations contained subpopulations, which had nonsense mutations in genes, such as the PTS system mannose/fructose/sorbose IID component family protein gene (W264* change in all ITC-exposed populations at 7 to 12% frequencies; Fig. 3; Supplementary Table S2), or the conserved hypothetical protein under DR76_2342 locus tag gene (W105* change in ITCD20TC and ITCD20RC populations at 7.7% and 5.8% frequencies, respectively; Fig. 3; Supplementary Table S2), or in the DNA mismatch repair endonuclease MutH gene (E148* change in ITCD14TC at 9.5%, in ITCD144C at 8.3% and ITCD20TC at 33.2% frequencies, respectively; Fig. 3; Supplementary Table S2). The ITCD20TC and ITCD20RC populations harboured a small proportion of subpopulations with an additional W108R *mutH* substitution (at 7.9% and 6.2% frequencies, respectively; Supplementary Table S2). MutH is the endonuclease of the DNA methyl-directed mismatch-repair (MMR) pathway, which is a pathway critical for the avoidance of mutations and maintenance of replicative fidelity. MMR defective bacteria have reduced ability to repair DNA damage, and are thus more prone to develop and accumulate mutations, including those conferring antibiotic resistance^31^. These mutations only existed in a small fraction of the population, however, and it is unknown whether finally they would become fixed in a population.

The key mechanisms of LVX resistance are chromosomal mutations that alter the A and B subunits of the target proteins, DNA gyrase (encoded by *gyrA* and *gyrB*) and topoisomerase IV (encoded by *parC* and *parE*); decreased intracellular drug accumulation due to increased drug efflux (mutations in *acrRAB*, *marRAB* and *soxSR* operons)^32^; or changes in outer membrane proteins (such as OmpF, OmpC, OmpD and OmpA)^33^. In our study, the population that was evolved under LVX selection carried a point mutation in the *gyrA* gene at a frequency of 100% on days 14 and 20 (Fig. 3; Supplementary Table S2). The nonsynonymous G to A substitution resulted in a S83L amino acid change, which is the most frequent mutation observed in quinolone-resistant *E. coli*^34^. This was a unique mutation, which occurred only in LVX-exposed whole populations (including the revived cultures). In addition, all the LVX-exposed populations uniquely possessed a *parC* G78D nonsynonymous substitution (LVXD14TC at 12.7%, LVXD14RC at 21.4%, LVXD20TC at 18.2% and LVXD20RC at 13.3% frequencies; Supplementary Table S2). The G78D change was an amino acid position (amino acid positions numbered according to *E. coli*) also known to be associated with LVX resistance in both Gram-positive and Gram-negative bacteria, although reports on this change are less frequent than changes in S80 or E84 of *parC*^35^. LVXD20TC also had other mutations occurring in the genes specific to quinolone resistance, such as nonsynonymous A426V, R432S substitutions and a 3 nt insertion in coding (1373/1893 nt) region of *parE*, as well as a 4 nt insertion in the coding (34/1089 nt) region of *ompF* gene (Fig. 3; Supplementary Table S2). The role that reduced expression, or the complete absence, of the OmpF porin plays in conferring fluoroquinolone resistance has been reported previously^36,37^. Our results indicated that decreased susceptibility and resistance to LVX in the LVX-exposed polymorphic culture were associated with multiple mutations in the *gyrA*, *parC, parE* and *ompF* genes. It is worth noting that LVXD20TC had a F36C nonsynonymous change in *mutS* gene (9.2%; Fig. 3; Supplementary Table S2). MutS is another protein of the MMR system, which recognizes and binds to mispaired nucleotides and enables the further action of the MMR machinery. It was shown that, as a result of *mutS* F36A substitution, MutS failed to recognize the mismatch site thus failing to initiate the repair^38^. In addition, LVXD20TC had elevated numbers of mutations (Fig. 2), perhaps, as a result of this *mutS* F36C modification. The LVX-exposed populations also had an increased number of insertions and deletions (indels). Interestingly, LVX-subjected populations also had several nucleotide insertions in the *yhjK* (LVXD20TC and LVXD20RC) and *yhjH* (LVXD14TC, LVXD20TC and LVXD20RC) genes (Fig. 3; Supplementary Table S2). YhjK is a predicted c-di-GMP phosphodiesterase (PDE), while YhjH is a c-di-GMP PDE involved in regulation of the switch from flagellar motility to sessile behaviour. Deletion of the *yhjH* gene and an insertion in *yhjH* gene was shown to impair the swimming motility of *E. coli*^39^ and contribute to the transition from planktonic to biofilm lifestyle. The 6 nt deletion in coding region (727–732/2706 nt) of *malT* gene, is also noteworthy (Fig. 3; Supplementary Table S2), perhaps as a modification to facilitate the adaptation of *E. coli* to nutrient limiting environment in chemostat^40^.

## Discussion

The potential for the development of resistance to an antimicrobial is an important factor in determining its suitability for therapeutic or environmental applications. We have previously shown that the repeated exposure of *E. coli*, *P. aeruginosa, S. aureus* and methicillin-resistant *S. aureus* to sub-inhibitory levels of ITC during serial passage of batch cultures did not generate ITC resistance^3^. In the present study, we used selection/enrichment chemostat culture to generate mutants with decreased susceptibility to ITC, coupled with WGS to identify those mutations underpinning resistance. Antimicrobial resistance may develop and increase *via* the gradual accumulation of multiple mutations^41^. To study this, we gradually challenged the evolving population in the chemostat with antimicrobials. Three separate enrichment continuous cultures of drug-susceptible *E. coli* ATCC 25922 were operated for 20 days to select for ITC and LVX-resistant mutants, which were then compared with selection-free culture. We measured the resistance using the MIC metric – resistance occurs when the population is able to grow in the environment of higher concentration of an antimicrobial^25^.

Planktonic chemostat populations grown without added selection pressure and with ITC selection pressure yielded populations with no altered susceptibility towards either ITC or LVX (Fig. 1a, b; Supplementary Table S1). These results indicate that ITC did not cause the emergence of resistance to itself, or the emergence of cross-resistance to the antibiotic LVX. However, attention should be given to the fact that there can be a possibility of population-level variability in terms of resistance, as only a minute fraction of the population was sampled. Further, ITC at 2 × MIC dose was able to eradicate a 10^20^ cfu ml^−1^ load of bacteria (Fig. 1b), a concentration much higher, for example, than the bacterial concentration in wound fluid allowing the progression of wound healing (<10^6^ cfu ml^−1^)^42^. This signifies that ITC may be suitable to disinfect heavily contaminated sites without risk of the facile emergence of resistance, even at very high cell densities. Nevertheless, we should note here that while ITC could eliminate a “weakened” population which was under the gradually increasing ITC stress over a long time period, it could not eliminate the resurrected ITC stress-“rested” population at the finish of this evolution experiment (Fig. 1b). On the other hand, the derivative chemostat culture, grown under LVX selection pressure, rapidly gained resistance towards LVX (MIC of LVX increased 64-fold by day 20), but no cross-resistance was observed towards ITC (Fig. 1c; Supplementary Table S1). LVX also failed to eradicate the bioburden at a 3 × MIC dose. Complete genome sequencing of these populations provided information about the mechanisms underlying the acquired phenotypes.

ITC is a biocidal mixture of reactive oxygen and iodine species. These reactive species can impair diversity of cellular macromolecules in bacteria and thus trigger protective oxidative stress responses towards these stressors. Moreover, these species alone can induce resistance-promoting responses in bacteria. The oxidative agents also promote the expression of multidrug efflux systems, likely to mitigate the impact of these stressors. Likewise, antioxidant mechanisms can be involved in response to exposure of ROS-based antimicrobials. Hence, oxidative stress responses may contribute to antimicrobial resistance in several ways^43^. Poole has reviewed, in detail, the evidence supporting links between the oxidative stress response and antimicrobial resistance in bacteria, and summarized the mechanisms of oxidative stress-induced resistance^43^. These include, amongst others, the redox-responsive regulator SoxSR of multidrug efflux system AcrAB-TolC in *E. coli*; e.g. reactive agents can cause direct mutations leading to constitutive *soxS* expression and elevated *acrAB* expression and, thus, antimicrobial resistance. We examined the genome sequences of ITC-exposed populations (Supplementary Table S2) for significant (nonsynonymous, nonsense) nucleotide variations, including those involving amino acid change or amino acid sequence termination, and compared with stress-inducible antimicrobial resistance mechanisms (Table 1 from reference)^43^. We could not identify any determinants, which could have contributed to oxidative (or stress in general) response-mediated resistance of the ITC-exposed culture to ITC and/or LVX.

On the other hand, cross-resistance between differing antimicrobial agents may arise when they act on the same target, access to their specific targets by a common way, or initiate the cell death by a common pathway. As a result, resistance to one antibacterial agent co-occurs with resistance to another agent^15,44^. LVX is a member of the fluoroquinolone class of antibiotics with broad antimicrobial activity against clinically relevant bacteria causing respiratory, skin and genitourinary tract infections. Following regulatory approval in the mid-1990s, LVX became one of the most widely prescribed antibiotics in the world^45^. The heavy prescription of LVX has led to increased resistance. LVX resistance is thought to be a result of gradually acquired changes, and several mutations are required to produce a high level of resistance^46^. In Gram-negative bacteria, resistance mutations firstly occur in *gyrA*, with “hot spots” for mutation at amino acid positions 83 and 87. Once this initial mutation has reduced the susceptibility of resistant bacteria to LVX, additional mutations in target genes *parC*, *parE*, or else, mutations causing reduced drug accumulation, can further augment the resistance^47^. Indeed, in our study, the day 14 LVX-exposed population was entirely composed of *gyr*A S83L mutants (occurrence 100%), while a proportion of the population also carried the *parC* G78D mutation (Supplementary Table S2). By day 20, the LVX-exposed population was further enriched with *parE* and *ompF* mutants (Fig. 3; Supplementary Table S2). These results indicate that, the acquisition of high level LVX resistance (e.g. a 64-fold increase in MIC) was related to mutations in genes encoding the three protein targets and mutations in uptake protein. In general, combining chemostat and WGS was a productive approach to generate, follow and understand the evolution of LVX resistance in *E. coli* ATCC 25922. ITC-exposed populations, by contrast, did not possess any common mutations conferring LVX resistance (Supplementary Table S2). In populations with the size described in these experiments, spontaneous mutations are virtually certain to occur at any position in the genome, including in the target genes of LVX. The fact that these mutations were not selected under ITC-selection pressure means that they were not (sufficiently) beneficial under ITC selection.

The MMR pathway safeguards the genome by correcting the base mismatches arising as a result of replication errors^48^. Recent studies suggest that MMR may also be important in the response to oxidative DNA damage^49^. Defects in MMR result in increased rates of mutations in organisms ranging from bacteria to humans. MMR-defective bacteria are considered to be “hypermutable” and to express a “mutator” phenotype (“mutator” strains). Mutator phenotypes, in general, result from alterations in genes coding for DNA repair enzymes (*mutS*, *mutL*, *mutH* and *mutU*). The genes affected in the studied mutator strains are *mutS*, *mutL*, *mutH* and *mutU* in decreasing order^50^. The *E. coli* ATCC 25922 population, which was grown under ITC selection, harboured a nonsense mutation in *mutH* gene on day 14 (detected in thawed culture (ITCD14TC) at a frequency of 9.5% and in 4 °C culture (ITCD144C) at a frequency of 8.3%) and on day 20 [detected at a frequency of 33.2% in thawed culture (ITCD20TC), but not in a revived culture (ITCD20RC)]. The LVXD20TC population also harboured the F36C nonsynonymous change in *mutS* gene at a frequency of 9.2% (Fig. 3; Supplementary Table S2). The ITCD20TC and LVXD20TC cultures, indeed, had an elevated number of mutations by comparison to the other samples (Fig. 2); however, we cannot definitively ascribe this to *mutH* or *mutS* mutations. These mutations only represented a small fraction of the total; but to be able to produce a mutator phenotype they must be genetically dominant. At present, there are mixed views about the link between hypermutation and emergence of antimicrobial resistance^50^. Schaaff and co-workers (2002) found that vancomycin resistance could be reached much more quickly in a mutator background (*mutS* knockout mutant) versus nonmutator (5 versus 19 passages, respectively)^51^. However, in our study during the timeline of the selection experiments, the ITC-exposed population did not develop elevated MIC values for ITC and LVX (Fig. 1b; Supplementary Table S1), while the LVX-exposed population showed elevated MIC values towards LVX (which was due to other primary mutations rather than *mutS*), but not toward ITC. These mutations may have appeared too late in the selection to become common in the populations. On the contrary, O’Neill and co-authors (2002) have concluded that stable hypermutation or transient increases in mutation frequency were probably not involved in the emergence of antibiotic resistance in *S. aureus*, despite the fact that a *mutS*-inactive *S. aureus* could have a 78-fold higher mutation frequency compared to the wild type strain^52^.

The overall conclusions of this study are that we were unable to generate *de novo* resistance to ITC in a drug-sensitive *E. coli* ATCC 25922, despite 20 days of selective evolution, and, thus, we could not identify mutations that would have yielded ITC resistance. Mutations in the gene of DNA mismatch repair endonuclease MutH may be implicated in elevated mutation numbers and result in mutator phenotype, thus contributing to antimicrobial resistance; however, these mutations did not become prominent in the evolved ITC-exposed population. By contrast, resistance to LVX quickly built up in the *E. coli* ATCC 25922 culture exposed to increasing concentrations of LVX, apparently driven by mutations in the genes coding for the target proteins (GyrA, ParC, ParE) and reduced drug accumulation (OmpF). Judging from the example of LVX, combining experimental evolution and whole-genome sequencing can provide a deeper understanding of the principles of how resistance emerges under different selective pressures.

The lack of emergence of facile resistance to ITC in *E. coli* ATCC 25922, and the lack of cross-resistance to LVX are encouraging with respect to the potential for applications of ITC as a biocidal agent in a variety of settings. An understanding of the possible mechanisms, if any, of ITC resistance, however, requires further longer-term investigations. The dynamics and determinants of potential ITC resistance should also be tested in numerous parallel cultures of a range of other bacteria, and the potential for cross-resistance studied on a range of important antibiotics.

## Materials and Methods

### Bacteria, growth media and antimicrobials

The parental strain used in this study was the drug-susceptible *E. coli* ATCC 25922. All the other cultures were derivatives generated by means of chemostat culturing. The lyophilized strain was resuscitated on fresh Lennox agar (Sigma-Aldrich) and the growth from a single colony was transferred into lysogeny broth (LB; Sigma-Aldrich). This single colony suspension was used to inoculate three parallel chemostat vessels in order to ensure that the parental strain utilized throughout the continuous culturing was genetically homogeneous at the start. The continuous cultures were grown in quarter-strength LB medium to ensure nutrient limitation. The developed cultures were stored at −20 °C in 20% glycerol.

LVX (Fluka) stock solution was prepared from powder in sterile deionized water (dH_2_O) and was filter-sterilized; ITC stock solution was prepared as previously described^3^. Briefly, to prepare 1% stock solution of ITC, hydrogen peroxide, potassium iodide and potassium thiocyanate aqueous solutions were mixed at 1:1:1 (v/v/v) ratio. Stock solutions of ITC and LVX were kept at 4 °C and reused for the duration of the experiments.

### Chemostat configuration and operation

A schematic diagram of the chemostat setup is illustrated in Supplementary Fig. S2 and was based on a design published previously^23^. Continuous cultures were performed in glass culture vessels (200 × 45 mm) with 100 ± 2 ml working volume equipped with the medium break, by which the media and the air were supplied. The oxygen was delivered into the vessel by pumping filter-sterilized air at a rate of ca. 100 ml min^−1^. The cultures were mixed by bubbling with sterile air. Medium flow from the reservoir to the culture vessel was controlled by a peristaltic pump (Watson-Marlow 505U). The working volume of the chemostat was fixed by the height of the central overflow tube. Fresh feed was introduced at constant flow rate and culture waste was removed at the same rate according to hydraulic principles. Chemostats were operated at a dilution rate of 0.4 hr^−1^. This rate of dilution will result in a generation time of 1.73 h per generation^53^. Samples were taken using a sterile syringe attached to the sampling port. Chemostat vessels were submerged in a temperature-controlled water bath and operated at 37 °C.

Three individual vessels (designated as selection-free, ITC and LVX-selection chemostats) were brought to their working volumes and inoculated from a single colony suspension of *E. coli* to obtain a final cell concentration of 10^6^ cfu ml^−1^. Cultivations were started in batch mode for 24 h to reach culture saturation, and then switched to chemostat mode by initiating the feeding. Meantime, the MICs of ITC and LVX toward this isogenic pre-culture were determined, and these values were used to infer the initial antimicrobial concentrations to be added in the chemostat feeds. Subsequently, two media feeds were supplemented separately with ITC and LVX at their 0.5 × MIC (7.81 and 0.024 µg ml^−1^, respectively). Antimicrobial concentrations in the media feed were increased stepwise by 0.5 × MIC increments when OD_600_ of ITC and LVX-exposed cultures reached to the value recorded prior the addition of the antimicrobial. Each chemostat culture was continuously operated for 20 days (282 generations). Increases in the feed concentrations of ITC and LVX to 2.5 × MIC and 3 × MIC, respectively, were made in the respective feeds during the chemostat course. Daily 2 ml samples were taken from chemostat cultures and used to test for a variety of parameters, such as OD_600_ readings, viable cell counts, broth MIC determinations, culture preservation and WGS of mixed planktonic population samples.

### Culture density, cell number and minimum inhibitory concentrations

The cell density of 1 ml chemostat-derived culture was measured using a spectrophotometer (Spectronic 20 Genesys) at a wavelength of 600 nm. Viable cell number was determined by colony counts on antibiotic-free agar plates after appropriate dilutions in phosphate-buffered saline. MICs for LVX and ITC in quarter-strength LB were measured by following growth in 96-well plates, as described previously^3^. In short, 3 inocula from a daily chemostat sample were prepared by taking aliquots from a planktonic population sample and diluting to cell density of 10^6^ colony forming units per ml (cfu ml^−1^) in three separate tubes. These 3 inocula at 5 × 10^5^ cfu ml^−1^ final density were challenged with ITC and LVX at serial 2-fold dilutions in 96-well plates. Microplates were incubated in culture chamber at 37 °C over 24 h. The MIC was defined as the lowest concentration of antimicrobial agent preventing the appearance of turbidity. The median of the triplicate measurements were reported as an MIC. For each of the chemostat cultures, the MIC change was calculated as the ratio of the MIC at sampling day to the MIC of starting day. Chemostat-derived *E. coli* cultures were considered to be resistant to ITC and/or LVX when a sustained >4-fold MIC increase from initial MIC was noted^54^. The actual MIC values from triplicate measurements of isogenic pre-culture at day 0, chemostat introduced cultures at day 1 and unexposed, ITC-exposed and LVX-exposed cultures throughout 20 days are presented in Supplementary Table S1. The observed differences between the MICs of isogenic pre-culture at day 0 and chemostat introduced cultures at day 1 might exist due to environmental conditions.

### Whole-genome sequencing of chemostat-derived polymorphic populations and mutation calling

The focal populations for WGS were unexposed chemostat culture from day 1, 14 and 20, ITC-exposed culture from day 14 and 20, and LVX-exposed culture from day 14 and 20. Aliquots (1 ml) of frozen glycerol stocks of planktonic polymorphic population samples (and not single colonies) collected from three chemostat cultures were thawed and immediately pelleted for genomic DNA isolation [further referred as thawed culture (TC)]. The samples represented approximately 1% of the total population in the chemostat. Likewise, a small aliquot was used to revive the frozen samples *via* overnight growth in 1 ml LB at 37 °C and pelleted for gDNA extraction [further referred as revived culture (RC)]. We could not revive day 14 ITC-exposed culture as a result of the total clearance of bacteria by ITC (Fig. 1b), and the leftover of the ITC-exposed day 14 culture earlier used for OD, CFU, MIC measurements and later kept at 4 °C was used for gDNA extraction and downstream bioinformatics analysis instead [further indicated as 4 °C culture (4 C)]. gDNA was extracted using QIAamp DNA Mini Kit (Qiagen), DNA concentration was quantified by Qubit Fluorometer (Life Technologies) and DNA integration was assessed by 0.75% agarose gel electrophoresis.

The genomes were sequenced using the Illumina-based platform of the MicrobesNG service (http://www.microbesng.uk) to generate 2 × 250 bp paired-end reads. For fourteen sequenced samples (6 control, 4 ITC and 4 LVX), the average coverage (over the 5130767 bp of the reference genome) ranged from 53–200×. Reads were adapter trimmed using Trimmomatic and the quality assessed using in-house scripts combined with SAMtools, BEDtools, and BWA-MEM *via* MicrobesNG. These reads were then aligned onto the *E. coli* ATCC 25922 reference genome (accession no. CP009072) and putative mutations were identified with the BRESEQ re-sequencing pipeline. This pipeline can detect SNPs, small indels (≤20 nt), as well as structural variations, such as large indels, duplications, mobile element insertions or other rearrangements, in polymorphic population samples^55–58^. As the samples were “metagenomic”, that is to say, a mixture of DNA from different individuals, BRESEQ was run at polymorphism mode with default parameters. BRESEQ uses a Kolmogorov-Smirnov test for bias in the qualities of bases supporting the new variant, and Fisher’s exact test for bias in the strands of reads supporting the new variant, in both cases rejecting the mutation if it was biased at a p-value = 0.05 significance level. The variation in reads at a given position was classified as a true population polymorphism (as opposed to sequencing error) when ≥5% of the reads differed from the others for a given allele.

The initial lists of predicted mutations by BRESEQ were manually edited. Mutations in repeat regions may not be fully predicted due to limitations of short-read DNA sequencing data. For that reason, we identified the positions of repeat regions in the reference genome using NUCmer (alignment of multiple closely related nucleotide sequences in MUMmer software) and BLASTN (alignment of somewhat similar sequences in National Center for Biotechnology Information website) to perform self-comparison. SNPs and small indel mutations, which had a BLASTN match in several locations, as well as large indels within repetitive regions or genes with multiple copy numbers, were ignored to avoid any concern about inaccurate read alignment^57,59^. We also discarded the mutations that were present to 100% in all samples (which were likely to be the difference between the reference *E. coli* ATCC 25922 and the starting isolate), considering only the mutations that accumulated throughout the selection experiment. A circular genomic map for the genomes of unexposed, ITC-exposed and LVX-exposed final day (day 20) thawed cultures were plotted using the BLAST Ring Image Generator (BRIG) software^60^.

## Supplementary information

Supplementary Figures

Supplementary Tables

## Data Availability

Raw sequencing reads from thawed and revived samples have been deposited in the European Nucleotide Archive’s Sequence Read Archive (Accession No. PRJEB27402 and PRJEB27390, respectively). The authors declare that all other relevant data generated or analysed during this study are included in this published article and its Supplementary Information files.
