# Supplementary material for: Continuous culture of Escherichia coli, under selective pressure by a novel antimicrobial complex, does not result in development of resistance
Source: Sci Rep. 2019 Feb 20;9:2401. doi: 10.1038/s41598-019-38925-9 (PMC6382887; doi:10.1038/s41598-019-38925-9)

Continuous culture of *Escherichia coli*, under selective pressure by a novel antimicrobial complex, does not result in development of resistance

Lilit Tonoyan, Gerard T. A. Fleming, Ruairi Friel and Vincent O'Flaherty

Supplementary Figure S1. The pattern of mutation sharing between thawed (TC) and revived (RC) cultures depicted as Venn diagrams. The exception is the ITCD14 sample, which we could not revive, instead, the mutations from the ITCD14 culture kept at 4°C are shown (ITCD144C). Untreated populations are in grey, ITC populations are in yellow, and LVX are in blue.

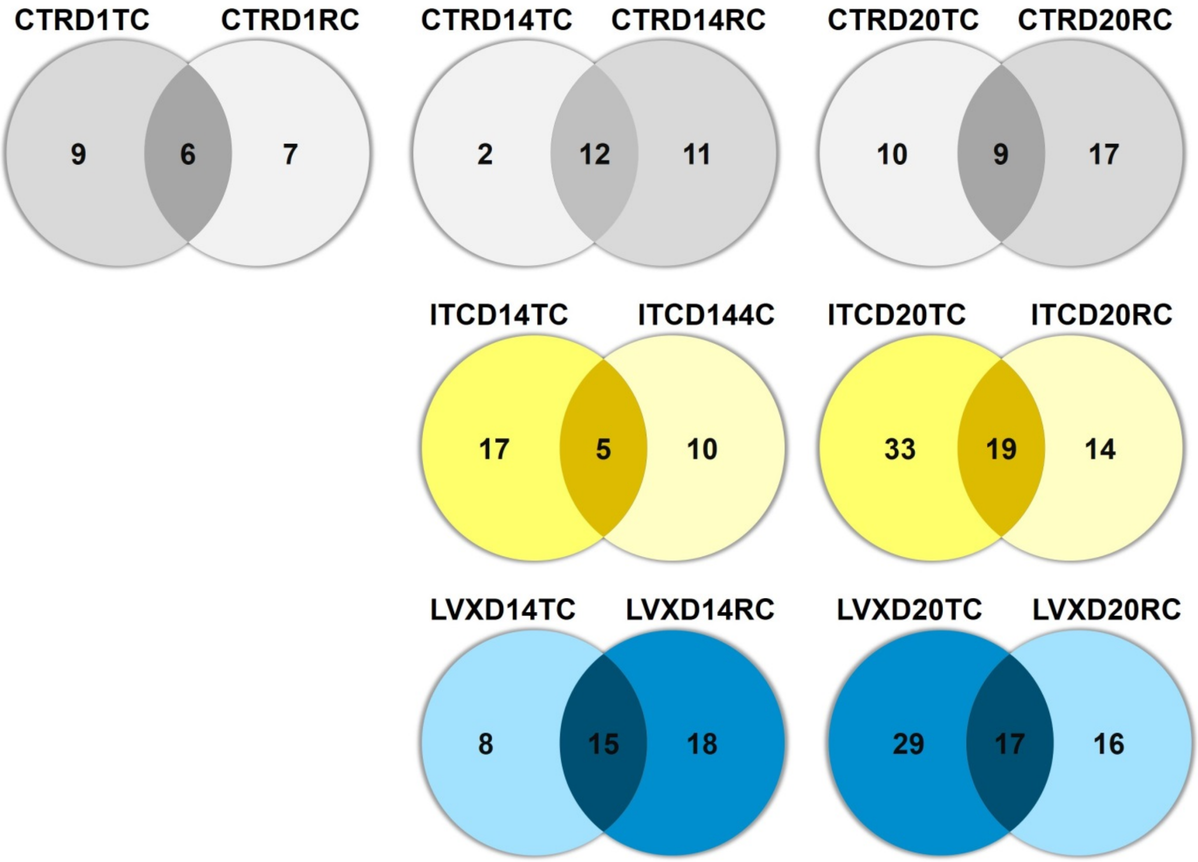

Supplementary Figure S2. Schematic representation of the chemostat experimental setup. The air supply system is coloured in blue, the media supply system is coloured in orange, while the outflow system is coloured in grey.

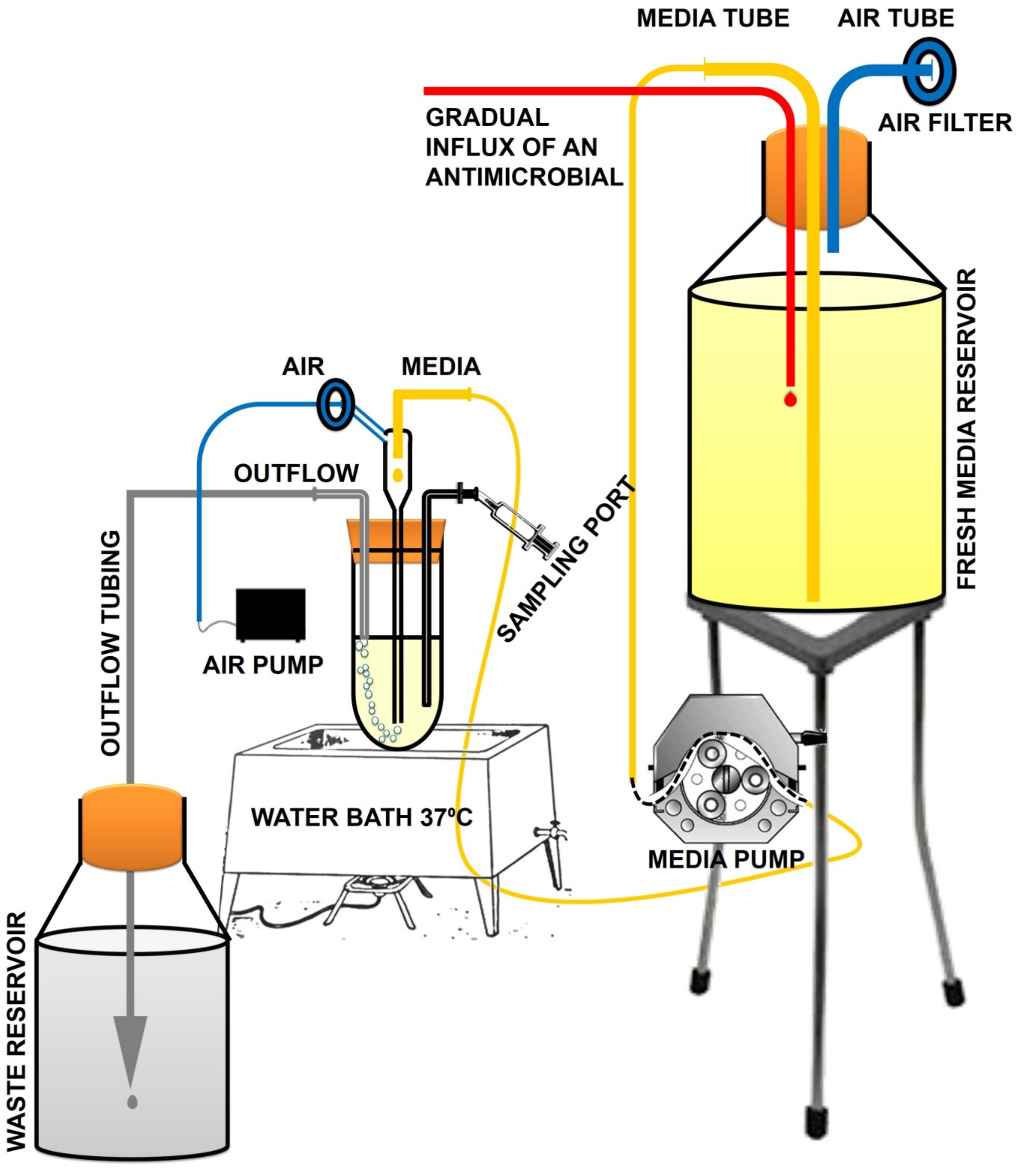

Supplement: Supplementary file 1 — Supplementary Figures [file 41598_2019_38925_MOESM1_ESM.pdf]
